# Supplementary material for: Structure-dynamics relationships in cryogenically deformed bulk metallic glass
Source: Nat Commun. 2022 Jan 10;13:127. doi: 10.1038/s41467-021-27661-2 (PMC8748940; doi:10.1038/s41467-021-27661-2)
Supplement: Supplementary file 1 — Supplementary Information [file 41467_2021_27661_MOESM1_ESM.pdf]

Structure-dynamics relationships in cryogenically deformed bulk metallic glass

Florian Spieckermann[1]*, Daniel Şopu[2,3], Viktor Soprunyuk[2,4], Michael B. Kerber[4], Jozef Bednarčík[5,6], Alexander Schökel[5], Amir Rezvan[2], Sergey Ketov[2], Baran Sarac[2], Erhard Schafler[4], and Jürgen Eckert[1,2]
*email: florian.spieckermann@unileoben.ac.at
[1]Department of Materials Science, Chair of Materials Physics, Montanuniversität Leoben, Jahnstraße 12, 8700 Leoben, Austria
[2]Erich Schmid Institute of Materials Science of the Austrian Academy of Sciences ,Jahnstraße 12, 8700 Leoben, Austria
[3]Institut für Materialwissenschaft, Fachgebiet Materialmodellierung, Technische Universität Darmstadt, Otto-Berndt-Strasse 3, Darmstadt D-64287, Germany
[4]Faculty of Physics, University of Vienna, Boltzmanngasse 5, 1090 Vienna, Austria
[5]Deutsches Elektronen Synchrotron (DESY), Notkestraße 85, 22607 Hamburg, Germany and
[6]P. J. Šafarik University in Košice, Faculty of Science, Institute of Physics, Park Angelinum 9, 041 54 Košice, Slovakia

I. SUPPLEMENTARY INFORMATION

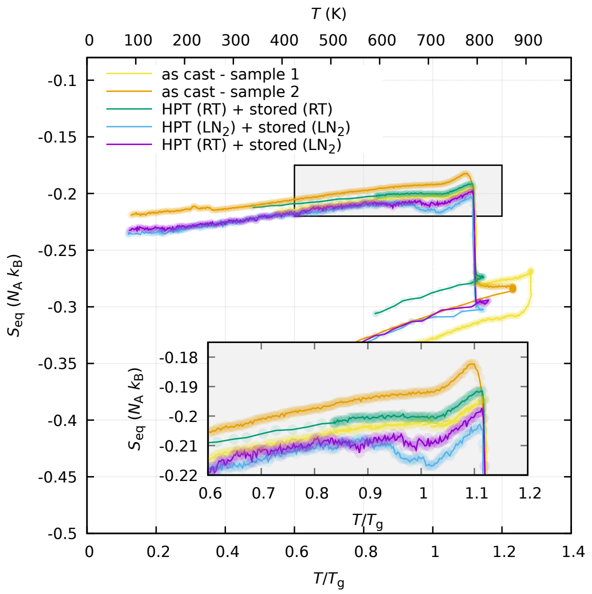

FIG. 1: Change in equivalent configurational entropy as determined by Equation 2. Each point is derived from a reduced PDF calculated from an X-ray diffraction pattern. The curves have **not** been shifted by an additive constant on the ordinate axis (S-axis).

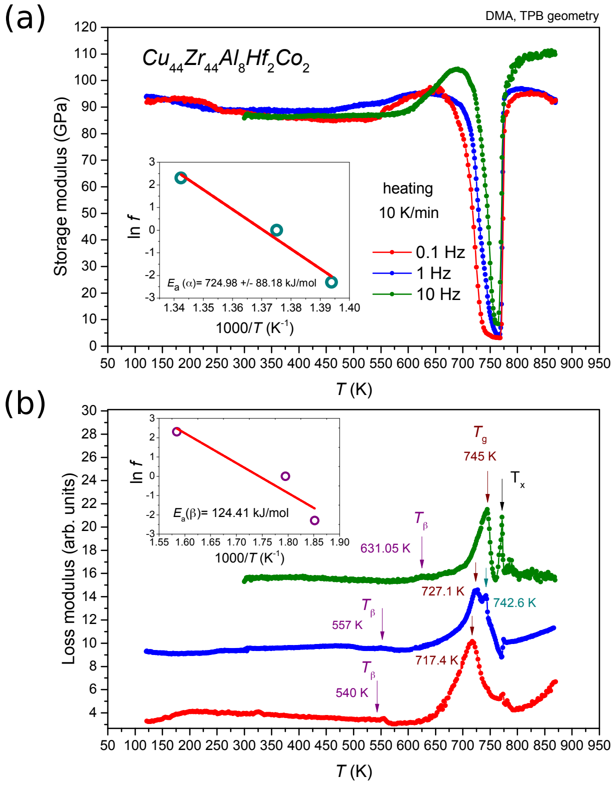

FIG. 2: DMA experiments probing the β- and α-transition. The temperature dependence of the storage modulus is shown in (a). The inset in (a) shows the the corresponding Arrhenius plot for the α-transition. The temperature dependence of the loss modulus is shown in (b). The inset in (b) shows the the corresponding Arrhenius plot for the β-transition.
