# Supplementary material for: Structure-dynamics relationships in cryogenically deformed bulk metallic glass
Source: Nat Commun. 2022 Jan 10;13:127. doi: 10.1038/s41467-021-27661-2 (PMC8748940; doi:10.1038/s41467-021-27661-2)
Supplement: Supplementary file 3 — Description of Additional Supplementary Files [file 41467_2021_27661_MOESM3_ESM.pdf]

Description of Additional Supplementary Files

File Name: Supplementary Movie 1
Description: shows the reduced pair distribution function G(r) for a sample deformed by high 5
pressure torsion (HPT) at 77 K and stored at 77 K.

File Name: Supplementary Movie 2
Description: shows the reduced pair distribution function G(r) for a sample deformed by high 8
pressure torsion (HPT) at room temperature (RT) and stored at 77 K.
